# The Concentration of ProAKAP4 and Other Indicators of Cryopotential of Spermatozoa Cryopreserved in Extender with *Holothuroidea* Extract Addition

**DOI:** 10.3390/ani12040521

**Published:** 2022-02-20

**Authors:** Alicja Kowalczyk, Elżbieta Gałęska, Anna Bubel

**Affiliations:** Department of Environment Hygiene and Animal Welfare, Wrocław University of Environmental and Life Sciences, Chełmońskiego 38C, 51-630 Wrocław, Poland; 113226@student.upwr.edu.pl (E.G.); anna.bubel@upwr.edu.pl (A.B.)

**Keywords:** reproduction, sperm, proAKAP4, extender, *Holothuroidea*, male

## Abstract

**Simple Summary:**

Scientists are constantly making efforts to determine the fertilization capacity of sperm after ejaculation. The present research proves that the application of *Holothuroidea* extract to the semen extender improves its cryostability, and has a positive effect on the biological quality of sperm after the freezing process. Moreover, it has been proven that the proAKAP4 biomarker can be successfully used to evaluate the effectiveness of the use of various extenders for semen cryopreservation.

**Abstract:**

The aim of this study was to determine the concentration of proAKAP4 and other indicators of cryopotential of spermatozoa cryopreserved in extender with *Holothuroidea* extract addition. Nine Holstein Friesian bulls, 3.5 years old, of known health status, were used for the study. The animals were kept and fed equally. Semen was collected once a week using an artificial vagina. The commercially available *Holothuroidea* extract was used as a supplement to the commercial extender (0, 2, 4 and 6 µL/mL) before the freezing/thawing process. The viability, motility, motion parameters, and acrosome integrity of the sperm were analyzed with (test) or without (control) extract samples. Furthermore, the concentration of the proAKAP4 biomarker in frozen sperm was assessed. It was shown that the addition of 4 and 6 µL of the extract may have a positive effect on the quality parameters of the sperm after thawing. The results indicate that extender supplementation with the above extract modulates (increases) the concentration of proAKAP4 in sperm at all tested levels. Additionally, this indicator has become helpful in identifying sperm of poor biological quality. Moreover, it has been proven that the proAKAP4 biomarker can be successfully used to evaluate the effectiveness of the use of various extenders for semen cryopreservation.

## 1. Introduction

With the advancement of reproductive biotechnology, it is obvious that the refinement of classical methods of semen evaluation and fertility prediction can bring significant economic savings to the livestock industry and local breeders. Therefore, scientists are constantly making efforts to determine the fertilizing capacity of sperm after ejaculation. Recently, advances resulting from basic research into male gametes have led to the development of a modern tool that may soon become a promising candidate for predicting the fertility of various animal species, as well as humans.

A4 kinase anchor protein (ProAKAP4) is a precursor polypeptide that must be transformed by live and motile spermatozoa in mature AKAP4, and its specific regulation during spermatogenesis seems necessary to increase the success rate under artificial insemination conditions [1,2]. Numerous studies have demonstrated the potential use of proAKAP4 as an appropriate biomarker of general semen quality in a clinical setting [2,3,4].

Moreover, ProAKAP4 concentrations have been shown to be correlated with the total and progressive motility of sperm cells in humans, stallions, and bulls [5,6,7]. It is clearly visible that sperm motility reflects the concentration of proAKAP4 and, thus, the measurement of this concentration provides more objective values than standard microscopic analysis (sperm are motile only at the time of analysis). Furthermore, various researchers have shown that proAKAP4 concentration measurements provide important information regarding the preservation of the biological quality of semen and can be used to predict male fertility [8,9].

The biological properties of *Holothuroidea* are still growing intensively, mainly due to the possibility of using these organisms in the food and pharmaceutical industries [10,11,12,13,14,15,16]. Due to the independent presence or absence of double bonds, hydroxy groups, acetates, keto- and other functionalities in their aglycones and numerous variants of sulfated or non-sulfated carbohydrate chains, *Holothuroidea* glycosides are a unique example of great biochemical diversity. As a result, these natural products have attracted the attention of several generations of chemists and biologists [11,12,13].

However, little is known about the use of proAKAP4 concentration measurements in standard semen cryopreservation protocols to evaluate the effectiveness of using extenders for long-term spermatozoa preservation. The proAKAP4 concentration assessment protocols published to date have not been standardized (they differ between laboratories), thus making it difficult to repeat them or apply them to other species. Although this marker is a promising tool for predicting the real quality of sperm, researchers still have a long way to go before they can be routinely used in the biological evaluation of spermatozoa. The aim of this study was to determine the concentration of proAKAP4 and other indicators of cryopotential of spermatozoa cryopreserved in an extender with *Holothuroidea* extract addition.

## 2. Materials and Methods

Nine Holstein Friesian bulls, 3.5 years old, with known health status, were used for the study. The animals were kept and fed equally. The bulls were kept at the insemination station (Malopolska Biotechnic Centre Ltd., Krasne, Poland) throughout the study. The studies were conducted in the period from March to May, for 10 weeks. It should also be noted that this study did not require the approval of the local ethics committee; all activities with the use of animals were routinely performed according to accepted standards at the insemination station.

### 2.1. Preparation of Macerate from Dried Holothuroidea Extract

The commercially available *Holothuroidea* extract (*A. japonicus,* Swanson, Frago, ND, USA) was taken from the cellulose shell. Then, 790 mg of powder was weighed and placed in glass tubes containing 10 mL of water-glycerin solution (40%/60% *v*/*v*). The tube was mixed with a vortex (AAA) for 5 min, then placed in a water bath and incubated for 15 h at 42 °C, pending further analysis.

### 2.2. Preparation and Initial Analysis of Sperm

Semen was collected from each male once a week (for 10 weeks) using an artificial vagina and a hydraulic phantom. The semen (in a graduated tube) was placed immediately after collection in a water bath (temp. 36 °C), where it awaited the preliminary evaluation.

Initial evaluations included mass motility, total motility, and sperm viability. Samples showing >55% viable and motile cells were qualified for further procedures.

### 2.3. Supplementation with an Extract

After an initial evaluation of semen quality, the ejaculates were pooled to eliminate individual differences between the males, then diluted to a final concentration of 80 × 10^6^ sperm/mL with a commercial extender (Optixcell, IMV, L’Agile, France) and divided into four equal samples. In the control (C) without additive, the test samples contained 2, 4 and 6 µL of extract. After being merged with the extract, the samples were gently mixed and subjected to detailed analysis.

Semen was automatically packed (Bloc Machine FIN, IS 4, France) into polyvinyl chloride (PVC) straws (0.25 mL) (Biovet, France), which were filled and equilibrated for 3 h at 4 °C. After equilibration, the straws were frozen in liquid nitrogen vapor using a computer-controlled automatic freezer from 4 °C to −15 °C at the rate of −3 °C/min and from −15 °C to −80 °C at the rate of −10 °C/min (IMV Technologies, Leger, France) [14].

After reaching −80 °C, semen straws were plunged into liquid nitrogen and packaged in plastic goblets for 24 h in the liquid nitrogen container. The straws were thawed in a water bath at 38 °C for 20 s and then examined to evaluate the quality after thawing [14].

### 2.4. Assessment of Sperm Viability

The double stain SYBR-14 with propidium iodide (L-7011 LIVE/DEAD Sperm Viability Kit; Invitrogen, Molecular Probes, Barcelona, Spain) using a flow cytometer was applied (CytoFlex Beckman Coulter, B3-R1-V0, Brea, CA, USA). For this purpose, 50 µL of thawed semen was measured (37 °C for 20 s) and 940 µL NaCl (0.9%) and 5 µL SYBR-14 were added. The whole was thoroughly mixed and then incubated (36 °C for 10 min) without light access. Subsequently, 5 µL of PI was remixed and incubated for 3 min without light, followed by a test [15].

### 2.5. Assessment of Sperm Motility

Mass motility was examined in 20 µL of semen, which was placed on a pre-warmed slide without any cover slip and analyzed under a microscope (Nikon E 200, Minato, Tokio, Japan) equipped with phase-contrast optics (100×) [10]. The mass motility was scored into four scales: + no motion, ++ free spermatozoa moving without forming any waves, +++ vigorous motion with moderately rapid waves, ++++ very rapidly moving waves.

Total sperm motility and progressive motion were examined using a sperm class analyzer (SCA, version 5.1, Microptic, Barcelona, Spain), and a light microscope (Nikon Eclipse E200). Just prior to analysis, semen was diluted 1:10 in a warm (25 °C) physiological solution (sodium chlorate 0.9%). Then, 2 μL of the prepared sample was placed in a Leja 4 analysis chamber (Leja Products B.V., Nieuw-Vennep, Holland) of 20.0 μm thickness. The slide was placed on a stage warmer (38 °C). A minimum of 500 cells were evaluated, and depending on sperm concentration, five analyses were performed per sample [15].

### 2.6. Assessment of Acrosome Integrity

Acrosome integrity was evaluated by propidium iodide (PI) and fluorescein isothiocyanate-conjugated Pisum sativum agglutinin (FITC-PSA), respectively. This association divides sperm populations into two groups: intact acrosome (IA) and damaged acrosome (DA). The procedure was performed with 200,000 cells diluted in Sp-Talp, stained with PI (0.5 mg/mL NaCl 0.9%) and FITC-PSA (FITC-PSA L-0770, Sigma, 100 μg/mL in sodium azide NaN 3 solution at 10% in DPBS). The samples were analyzed by flow cytometry after 10 min. A minor modification to the methodology was applied to the previously published procedure [15].

### 2.7. Assessment of ProAKAP4 Concentration

The measurement of ProAKAP4 concentrations was performed using a commercial ELISA kit according to the manufacturer’s instructions (4BioDx, Lille, France). In brief: first, the sperm were thawed at room temperature for 45 s. The sperm were then resuspended by gentle agitation of the sample. The sperm volume containing a minimum of 1 million spermatozoa was pipetted and diluted with 3 volumes of RPMI-1640 medium (Thermo Fisher, Waltham, MA, USA) in a 15 mL conical tube. The diluted sperm was spun for 10 min at 700× *g*. The supernatant was collected and discarded and 100 μL of RPMI-1640 medium (Thermo Fisher, Waltham, MA, USA) was added to the pellet of spermatozoa. The spermatozoa were resuspended in RPMI-1640 medium (Thermo Fisher, Waltham, MA, USA) by gentle agitation.

Subsequently, drop by drop, 2 mL of paraformaldehyde was added at 3% and the solution was vortexed at the lowest speed. Spermatozoa were incubated for 2 h at room temperature in the dark. The spermatozoa were pelleted by centrifugation at 500× *g* for 10 min.

The supernatant was then collected and discarded. A total of 100 μL of PBS was added and fixed spermatozoa were resuspended by gentle agitation. Progressively, 1 mL of PBS was added. This step was repeated twice. After the second centrifugation, the supernatant was discarded, and 200 μL of PBS was added; then, the spermatozoa pellet was resuspended again in RPMI-1640 medium (Thermo Fisher, Waltham, MA, USA) by gentle agitation.

The monoclonal anti-proAKAP4 (clone 6F12) (ref. 4BDX-1701, 4BioDx, France) was diluted in PBS containing 0.3% (weight/volume; 1 μg/mL) of Triton X-100. A total of 100 μL of the monoclonal antibody anti-proAKAP4 was added to the spermatozoa pelleted and incubated at room temperature for 45 min. After that, 1 mL of PBS was added, and the solution was spined at 500× *g* for 5 min and resuspended with the secondary antibody dilution (100 μL).

The secondary antibody was diluted in PBS added with 3% of Triton X-100 at a final concentration of 0.5 μg/mL and incubated for 45 min at room temperature in the dark. The solution was spined, the supernatant was discarded and 400 μL of PBS was added. The sample was loaded and made an acquisition of FS/SS (fluo excitation at 488 nm/emission at 530 nm).

To avoid aggregate formation and specifically analyze separated sperm cells separated from the cellular debris, a specific gating strategy was developed using the example of Blommaert [5]. The second selection for gating on FCS and FITC staining with an area-by-width parameter was performed after single-cell gating from aggregates to gate only single proAKAP4-stained cells (thus, only single events were analyzed). No overlap was observed between the spectrum of the secondary antibody and proAKAP4.

### 2.8. ProAKAP4 ELISA Assays

Semen was first thawed on ice. Then, 50 µL sperm suspension was added to 450 µL of lysis buffer and proceeded with ELISA quantification using the proAKAP4 ELISA kit (4BioDx, 4VDX-18K3, France) [5]. The samples and standards were processed using commercial buffers and according to the manufacturer’s instructions (4BioDx, 4VDX-18K3, France). In brief, 55 µL of semen was added to each well of the plate precoated with an antibody with proAKAP4 and incubated for 1.5 h. After the first washing, the horseradish conjugated antibody was incubated for 1 h. Following a second washing step, the substrate solution was then added to each well and incubated for 30 min. The intensity of the color was proportional to the proAKAP4 concentration in the sperm samples. The color reaction was stopped using a stop solution for 1.5 min and the absorbance was measured by spectrophotometry at 450 nm (The Thermo Scientific™ Multiskan™ Sky Microplate Spectrophotometer, Waltham, MA, USA). A standard curve was determined for concentrations of proAKAP4 between 4.7 and 150 ng/mL. The results of the proAKAP4 concentrations in the bull semen were expressed in ng/mL.

### 2.9. Statistical Analysis

Data are presented as mean (±) standard error of the mean (SEM). Analysis of variance (ANOVA) was used to assess differences between the stages of *Holothuroidea* extract supplementation on all characteristics of semen. When the F ratio was significant (*p* < 0.05), Duncan’s multiple range test was used to compare the means of treatment. Statistical analysis of the results was performed using Statistica 12.0 (StatSoft, Kraków, Poland). The study was repeated 10 times.

## 3. Results

The mean value of total sperm motility in fresh sperm obtained in the samples before qualification for further procedures was 78.41%; the mean of sperm mass motion was estimated at the average level (++), while the percentage of live sperm in the ejaculate was 69.04% on average.

A computer-assisted analysis of sperm motion parameters (Table 1) showed that, in semen containing 4 and 6 µL of the extract, the percentage of progressive motion sperm cells was significantly higher (*p* < 0.05) than in the control group. Supplementation with the *Holothuroidea* extract also had a positive effect on other parameters of cell motion, i.e., VCL, VAP, VSL, and LIN. The indicated parameters were significantly higher (*p* < 0.05) in the groups supplemented with 6 µL of the extract compared to the control group (without the addition). There were no significant differences between the groups in the ALH parameter.

The results of the analysis of acrosome integrity and sperm viability are presented in Table 2. It was observed that, with the increasing concentration of *Holothuroidea* extract, the percentage of sperm with an intact acrosome also increased by 4.24%; 6.91% and 7.15%. A significant decrease (*p* < 0.05) in the percentage of damaged sperm was observed in the samples with the addition of 4 and 6 µL of the extract.

The mean sperm viability (Table 2) in the control group was lower than in the tested samples, with the addition of the *Holothuroidea* extract by 1.53%; 2.56% and 4.93%, respectively; however, only the samples containing 6 µL of the extract were significantly (*p* < 0.05) more viable than the control group. Similar results were observed for the percentage of dead (necrotic) sperm. In the test group containing 6 µL of the extract, significantly less (*p* < 0.05) sperm with damaged cytoplasmic membrane was recorded.

**Figure 1:**
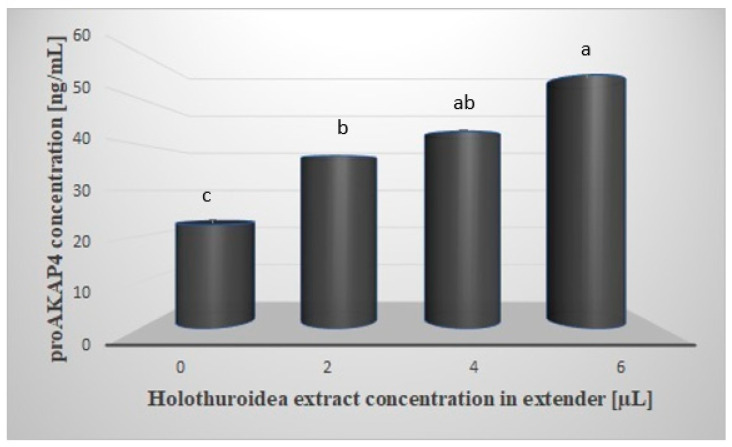
Mean concentration values of proAKAP4 in frozen/thawed sperm cells in an extender supplemented with *Holothuroidea* extract. Explanations: a, b, c: means with different superscript letters (between bars) differ significantly at *p* < 0.05. The values are expressed as mean ± SD. 0: control group without extract addition 2, 4, 6: concentration of extract [µL].

**Figure 2:**
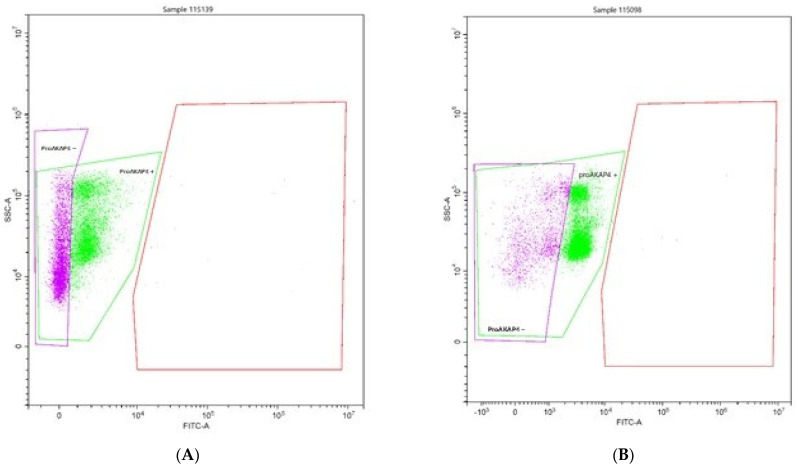
Histograms of two populations of spermatozoa were obtained by flow-cytometry. (**A**) sperm with poor quality. (**B**) sperm with good quality.

Figure 1 shows the mean concentration of proAKAP4 in the sperm samples tested. Spectrophotometric analysis showed a significantly higher (*p* < 0.05) concentration of proAKAP4 in all samples containing the extract. The mean concentration of proAKAP4 in the control group was 22.55 ng/mL and was lower by 14.94; 20.26 and 32.51 ng/mL, respectively, compared to the rest of the test groups. The highest concentration of proAKAP4 was observed in the group with the addition of 6µL of extract, at 55.06 ng/mL. A significant effect (*p* < 0.05) of the tested extract was also observed between the groups treated with 2 and 6 µL of extract (37.49 vs. 55.06 ng/mL) Figure 2 shows the apparent difference in the cytometric histogram between low-quality (A) and good-quality (B) semen.

Flow-cytometry analyses clearly delineate two populations of frozen/thawed spermatozoa. Spermatozoa proAKAP4 (+) or without proAKAP4 (−) are indicated. Spermatozoa that did not express proAKAP4 exemplified poor-quality semen with a concentration of 24 ng/10 M spz. (A) sperm with poor quality; (B) sperm with good quality.

## 4. Discussion

The biological activity of the ingredients contained in *Holothuroidea* (chondroitin sulphates, fucan sulphate, saponins, polysaccharides, fatty acids), has become the object of interest of many scientific disciplines [16,17,18]. *Holothuroidea* has been shown to have both antimicrobial, antifungal, antiprotozoal, antiinflammatory, anticoagulant, antitumor, antioxidant and antiviral potential [19,20,21]. At present, there are no studies related to the use of this natural product in andrology.

Our study showed that specific concentrations (4 and 6 µL) of *Holothuroidea* extract, when added to the extender, significantly (*p* < 0.05) improved sperm survival after the freezing process, preserved the acrosome structure and increased sperm motion parameters. The higher sperm motility may be the result of the polysaccharides contained in *Holothuroidea*, which are known as a source of sperm energy. Furthermore, the better results found in the integrity of the cytoplasmic membrane or acrosome may be the result of an antioxidant effect of this supplement. Oxidative stress (redox imbalance) has the potential to reduce viability as well as increase DNA fragmentation and may cause damage to the sperm acrosome during cryopreservation [14].

The proAKAP4 biomarker was used for the first time to evaluate the effectiveness of sperm freezing in various preservation environments in the described study. It is widely known that conventionally assessed parameters of the biological quality of semen are considered unsatisfactory because they do not correlate well with fertility, which causes problems with the prediction of breeding potential [22]. Recently, testing methods have been developed to directly recognize the relationship between sperm physiology and its functional features, and the proAKAP4 is presently a candidate as a key fertility marker [23].

In this study, the effect of *Holothuroidea* extract on the concentration of proAKAP4 in frozen/thawed sperm was analyzed. The results indicate that extender supplementation with the mentioned extract modulates (increases) the concentration of proAKAP4 in the sperm at all tested levels. It was also observed that an increase in the population of sperm cells with better motility parameters, higher viability and less acrosome damage was associated with a higher concentration of proAKAP4 in the tested semen. The group with high proAKAP4 concentrations (samples with the addition of 6 μL of the extract) are also the populations with higher progressive motility, less damaged cells, higher viability and higher straight-line velocity (μm/s) (VSL). These results are also in accordance with the results of Bastan and Akcay [13], which describe high-quality sperm traits in high-quality semen. In other studies, a positive correlation was found between the proAKAP4 level and total motility and progressive motility values in humans [6]. Previous studies performed in the stallion, ram, dog and bull frozen-thawed semen samples reported a positive correlation between the proAKAP4 levels determined by ELISA and CASA techniques in terms of total motility, progressive motility, LIN, VCL, and VSL [8,9,24].

It has been proved that *Holothuroidea* are sources of carbohydrate, among others, and these are known to be a valuable source of the energy needed to maintain sperm motility [25,26]. Current research shows the central contribution of AKAP4 to anchor the glycolytic enzymes (ProAKAP4 and the active AKAP4 are essential to localizing the glycolytic enzyme) that regulate sperm motility and hyper-motility and, hence, are necessary to produce the energy needed for a sustained motion of spermatozoa flagellum [8,9,24].

Although the routine use of the proAKAP4 biomarker in the evaluation of semen of various species is still in its infancy, the interest in maintaining a high level of proAKAP4 in sperm (after thawing), in the context of the fertilizing potential of sperm associated with long-term cell motility, continues to increase.

Additionally, this indicator has become helpful in identifying sperm of poor biological quality. Finally, a higher presence of the proAKAP4 is a result of better AKAP4 function, including the flagellum structure, motion, capacitation, and fertility.

## 5. Conclusions

The present research proves that the application of *Holothuroidea* extract to the semen extender improves its cryostability and has a positive effect on the biological quality of sperm after the freezing process. Moreover, it has been proven that the proAKAP4 biomarker can be successfully used to evaluate the effectiveness of the use of various extenders for semen cryopreservation.

## Figures and Tables

**Table 1:** Sperm motility after cryopreservation in an extender supplemented with *Holothuroidea* extract.

Parameters	Control Group (without Extract)	Holothuroidea Extract Treatments (Concentration, µL)		
	0	2	4	6
Progressive motility (%) (PM)	50.02 ^c^ ± 2.2	52.78 ^b,c^ ± 1.1	54.40 ^b^ ± 1.4	56.93 ^a^ ± 1.5
Curvilinear velocity (μm/s) (VCL)	64.15 ^b^ ± 2.4	65.94 ^b^ ± 1.6	67.39 ^a,b^ ± 1.2	68.06 ^a^ ± 1.4
Average path velocity (μm/s) (VAP)	47.29 ^b^ ± 1.9	48.85 ^a,b^ ± 2.0	49.21 ^a,b^ ± 1.7	53.33 ^a^ ± 1.6
Straight-line velocity (μm/s) (VSL)	42.77 ^b^ ± 1.2	44.51 ^a,b^ ± 2.1	45.91 ^a,b^ ± 1.2	46.56 ^a^ ± 1.7
Linearity (%) (LIN)	58.63 ^b^ ± 0.9	59.00 ^b^ ± 0.8	63.70 ^a,b^ ± 0.6	64.36 ^a^ ± 1.0
Amplitude of lateral head displacement (μm) (ALH)	2.62 ^a^ ± 0.1	2.59 ^a^ ± 0.1	2.55 ^a^ ± 0.1	2.4 ^a^ ± 0.2

Explanations: means with different superscript letters (a, b, c) in the same row differ significantly at *p* < 0.05. The values are expressed as mean ± SD.

**Table 2:** Acrosome integrity and viability of frozen/thawed sperm cells in extender supplemented with *Holothuroidea* extract.

Groups		Acrosome Integrity	Sperm Viability		
		Intact (%)	Live (%)	Early Apoptotic (%)	Dead (%)
Control group (without extract)		43.05 ^b^ ± 1.9	52.14 ^b^ ± 2.5	3.06 ^b^ ± 0.5	44.80 ^b^ ± 2.3
Holothuroidea extract treatments (concentration, µL)	2	47.29 ^a,b^ ± 2.2	53.67 ^b^ ± 2.5	1.41 ^b^ ± 0.7	44.92 ^b^ ± 2.6
	4	49.96 ^a^ ± 2.2	54.70 ^a,b^ ± 2.8	4.08 ^a,b^ ± 0.9	41.22 ^a,b^ ± 3.0
	6	50.20 ^a^ ± 2.4	57.07 ^a^ ± 3.1	11.09 ^a^ ± 1.1	38.71 ^a^ ± 3.2

Explanations: means with different superscript letters (a, b, c) in the same column differ significantly at *p* < 0.05. The values are expressed as mean ± SD.

## Data Availability

The datasets used during the current study are available from the corresponding author on reasonable request.
